# Water, Sanitation, and Cholera in Sub-Saharan Africa

**DOI:** 10.1021/acs.est.3c01317

**Published:** 2023-07-06

**Authors:** Mustafa Sikder, Aniruddha Deshpande, Sonia T. Hegde, Espoir Bwenge Malembaka, Karin Gallandat, Robert C. Reiner, Justin Lessler, Elizabeth C. Lee, Andrew S. Azman

**Affiliations:** † Department of International Health, Johns Hopkins Bloomberg School of Public Health, Johns Hopkins University, Baltimore, Maryland 21205, United States; ‡ Institute for Health Metrics and Evaluation, University of Washington, Seattle, Washington 98105, United States; § Department of Epidemiology, Johns Hopkins Bloomberg School of Public Health, Johns Hopkins University, Baltimore, Maryland 21205, United States; ∥ Center for Tropical Diseases and Global Health (CTDGH), Université Catholique de Bukavu (UCB), B.P. 285 Bukavu, The Democratic Republic of Congo; ⊥ Environmental Health Group, Department for Disease Control, Faculty of Infectious and Tropical Diseases, London School of Hygiene and Tropical Medicine, London WC1E 7HT, U.K.; # Department of Health Metrics Sciences, School of Medicine, University of Washington, Seattle, Washington 98105, United States; ∇ Geneva Centre for Emerging Viral Diseases, Geneva University Hospitals, Geneva 1205, Switzerland; ○ Division of Tropical and Humanitarian Medicine, Geneva University Hospitals, Geneva 1205, Switzerland

**Keywords:** population-level analysis, random forest, geographic classification, risk analysis, infrastructure access

## Abstract

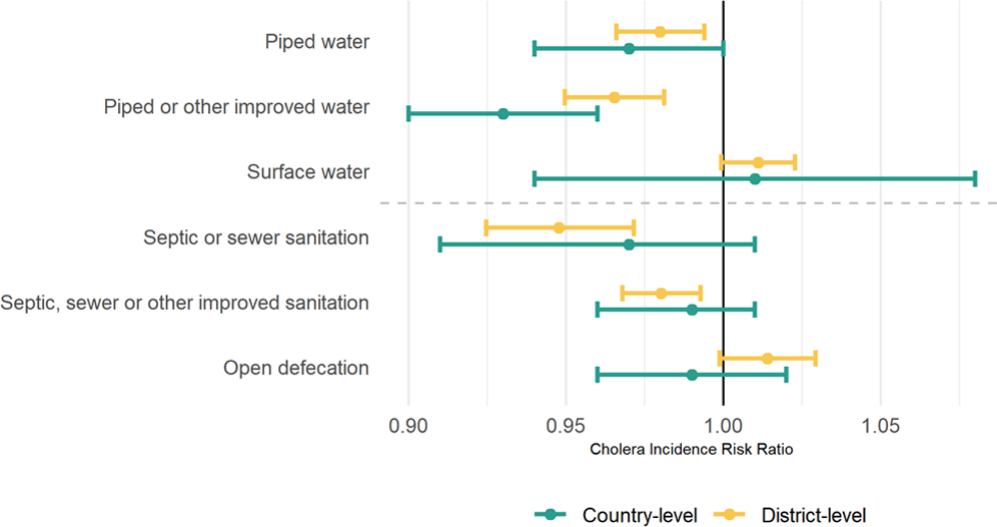

Improvements in water and sanitation should reduce cholera risk though the associations between cholera and specific water and sanitation access measures remain unclear. We estimated the association between eight water and sanitation measures and annual cholera incidence access across sub-Saharan Africa (2010–2016) for data aggregated at the country and district levels. We fit random forest regression and classification models to understand how well these measures combined might be able to predict cholera incidence rates and identify high cholera incidence areas. Across spatial scales, piped or “other improved” water access was inversely associated with cholera incidence. Access to piped water, septic or sewer sanitation, and septic, sewer, or “other improved” sanitation were associated with decreased district-level cholera incidence. The classification model had moderate performance in identifying high cholera incidence areas (cross-validated-AUC 0.81, 95% CI 0.78–0.83) with high negative predictive values (93–100%) indicating the utility of water and sanitation measures for screening out areas that are unlikely to be at high cholera risk. While comprehensive cholera risk assessments must incorporate other data sources (e.g., historical incidence), our results suggest that water and sanitation measures could alone be useful in narrowing the geographic focus for detailed risk assessments.

## Abstract 2

We quantified the relationship between high-resolution estimates of water and sanitation access and cholera incidence and assessed the utility of water and sanitation measures in identifying high-risk geographic areas in sub-Saharan Africa.

## Introduction

Access to safe water and sanitation are measured to assess progress toward the sustainable development goals (SDG). Safe water and sanitation reduce the risk of water-borne diseases, improve health, and are considered fundamental human rights.^1^ The joint monitoring programme (JMP), a collaboration between the World Health Organization (WHO) and United Nations Children’s Fund (UNICEF), defines improved drinking water sources as those that have the potential to deliver safe water, including piped water, boreholes, protected dug wells, protected springs, rainwater, and packaged water, and improved sanitation as those facilities designed to hygienically separate excreta from human contact.^2^

*Vibrio cholerae*, the bacteria that causes cholera disease, is primarily transmitted through contaminated food and water.^3^ While eliminating fecal contamination of water and food by *V. cholerae* should greatly reduce cholera risk, evidence documenting the impact of water, sanitation, and hygiene (WASH) on cholera in contemporary low- and middle-income settings remains limited. This is likely due to heterogeneity in WASH intervention implementation, and the gap between access and use of safely managed infrastructure. Two systematic reviews identified low and medium-quality studies that measured the impact of short-term WASH interventions on cholera incidence; the settings, study designs, and interventions were highly variable, and the estimated reduction in cholera incidence ranged from 0 to 88% across studies and interventions.^4,5^

Population-level studies examining the association of WASH infrastructure and/or behaviors (without an explicit intervention) and cholera risk found more consistent evidence that WASH-related exposures were positively associated with cholera risk. A systematic review of individual and household-level factors found that unimproved water sources and open container water storage increased the odds of symptomatic cholera, while household water treatment and hand hygiene decreased it.^6^ Another systematic review of 51 case–control studies found that eight WASH risk factors were associated with higher odds of cholera and five out of seven WASH protective factors were associated with lower odds of cholera, although 80% of the studies were evaluated to have medium or high risk of bias.^7^ Finally, a population-level analysis found that national estimates of access to improved water sources and improved sanitation only had limited predictive value in identifying endemic cholera countries.^8^

With current commitments to reduce cholera burden, including the Cholera Roadmap 2030,^9^ several countries are developing multiyear, multisectoral national cholera control plans. In developing these plans, countries must determine how to best utilize limited resources and seek an evidence base to help make these decisions. In this context, robust, quantitative evidence of the association between specific water and sanitation exposures and cholera risk can inform future decision-making on the geographic prioritization of cholera interventions.

In our study, we leverage estimates of water and sanitation services^10^ and suspected cholera incidence from sub-Saharan Africa^11^ to explore the association between water and sanitation infrastructure access and cholera risk at national and sub-national scales.

## Methods

### Data Sources

We used previously published mean annual incidence estimates of suspected cholera (referred to throughout simply as “cholera”) from 2010 to 2016 in 20 km × 20 km grid cells across sub-Saharan Africa excluding Botswana, Djibouti, and Eritrea.^11^ We obtained mean annual estimates originally made at the 5 km × 5 km grid cell level of two sets of four mutually exclusive and collectively exhaustive indicators of access—one set for drinking water and one for sanitation.^10^ Both the drinking water indicator set (piped water on or off premises, other improved facilities, unimproved, and surface water) and sanitation indicator set (septic or sewer sanitation, other improved, unimproved, and open defecation) collectively accounts for 100% of the population in the respective geographical area. The facility type classification scheme employed for assessing access to water and sanitation facilities in our analysis differed partially from the new JMP service level classifications that have been developed for monitoring the SDG targets. In our analysis, we grouped four putative protective measures: access to piped water on or off premises, access to “other improved” water, access to sewer or septic sanitation, and access to “other improved” sanitation. The remaining measures (reliance on unimproved (unprotected wells and springs) water, surface water (untreated from lakes, ponds, rivers, and streams), unimproved sanitation (unimproved latrines, buckets, hanging toilets), and open defecation) were considered as putative risk factors. We obtained 1 km × 1 km resolution population size estimates from the WorldPop Open Population Repository for 2010–2016. We conducted analyses at two spatial scales: country (*n* = 40) and second-level administrative unit level (*n* = 4146) with administrative boundaries based on the Database of Global Administrative Areas version 3.6.^12^

### Analysis

Our analyses included 40 countries in sub-Saharan Africa where both suspected cholera case incidence data and water and sanitation data were available. All water and sanitation measures were reported as percent of people with access to protective measures or reliance on risk measures. For the water and sanitation measures, we first calculated mean access/reliance across annual estimates from 2010 to 2016 and mean population counts across the same period; this period matched the period corresponding to the mean annual cholera incidence estimates. Then we calculated population-weighted country means for water and sanitation measures and annual cholera incidence. To quantify the association between mean annual cholera incidence and mean water and sanitation measures by country, we used univariate Quasi-Poisson regression with water and sanitation measures as linear predictors of cholera incidence and total population of the country as an offset term.

We aggregated water and sanitation and cholera measures at the second-level subnational administrative unit (*n* = 4146), hereafter “district”. We defined high cholera incidence areas as districts where >10% of the population or >100 000 people lived in a grid cell with a mean annual incidence rate > 1 per 1000 cases/year, following previous work.^11^ To study the relationship between district-level mean annual cholera incidence and water and sanitation measures, we used univariate Poisson generalized estimating equations (GEE) with country as a cluster variable and district population as an offset term. Estimates of risk ratios for exposures that are ordinal in nature can be challenging to interpret (e.g., risk ratio for access to improved water will include both those with “better” (piped water) and “worse” (unimproved water and surface water) access to water in the comparison group). Therefore, to have more interpretable relative risk estimates, we arranged the water measures as piped water, piped or “other improved” water, and surface water and sanitation measures as septic or sewer sanitation; septic, sewer, or “other improved” sanitation; and open defecation. The unimproved water and unimproved sanitation categories were excluded from the analysis since the comparison group will include both “better” (piped and other improved) and “worse” (surface water or open defecation) categories making estimates of the association difficult to interpret.

We then used random forest models to understand the potential predictive value of all water and sanitation measures combined for predicting suspected cholera incidence rates and for identifying high cholera incidence areas (e.g., identifying administrative units where incidence rates exceed a specific threshold). We conducted leave-one-district-out cross-validation to evaluate the model performance and then ran the model on the full data to understand the predictive importance of the water and sanitation measures. For the regression models, which had mean annual incidence as the dependent variable, model fit was judged by cross-validated root-mean-square error (cvRMSE). In classification models, meant to discriminate areas with high incidence from those without high incidence, model fit was judged by area under the cross-validated receiver operator characteristic curve (cvAUC). We performed oversampling with replacement from the minority class to address the class imbalance between high incidence areas (*n* = 309) and areas without high incidence (*n* = 3837) in each fold using previously published methods.^13^ To understand the relative importance of each water and sanitation measure within the models, we calculated the conditional permutation importance (CPI) matrix.^14^ In secondary analysis, we fit high incidence area classification models only including countries where at least one district was classified as high cholera incidence area (25 countries) and explored the performance of gradient boosting machine (GBM) models.

To match the varying spatial resolution of different raster layers we used the “resample” function from Raster package^15^ with the bilinear interpolation method, which uses a weighted average to calculate the new cell values. The data aggregation for the district- and country-level estimates were completed using the *Exactextractr* package^16^ with the “exact_extract” function by calculating the mean value of the cells coveted by the feature boundary. The generalized estimating equations were computed using *gee*,^17^ the random forest models were completed using the *randomForest*,^18^ variable importance were obtained using *permimp*,^14^ and GBM models were completed using *gbm*(19) packages in R. Code and data needed to reproduce primary analyses are available at the code repository (https://github.com/mustafasikder/wash_cholera).

## Results

We summarized eight water sanitation measures and cholera incidences at the country (*n* = 40) (Table S1) and district levels (*n* = 4146) from 2010 to 2016 along with suspected cholera incidence estimates from 2010 to 2016. These datasets are described in detail in their original publications.^10,11^

### Country Level

**Figure 1:**
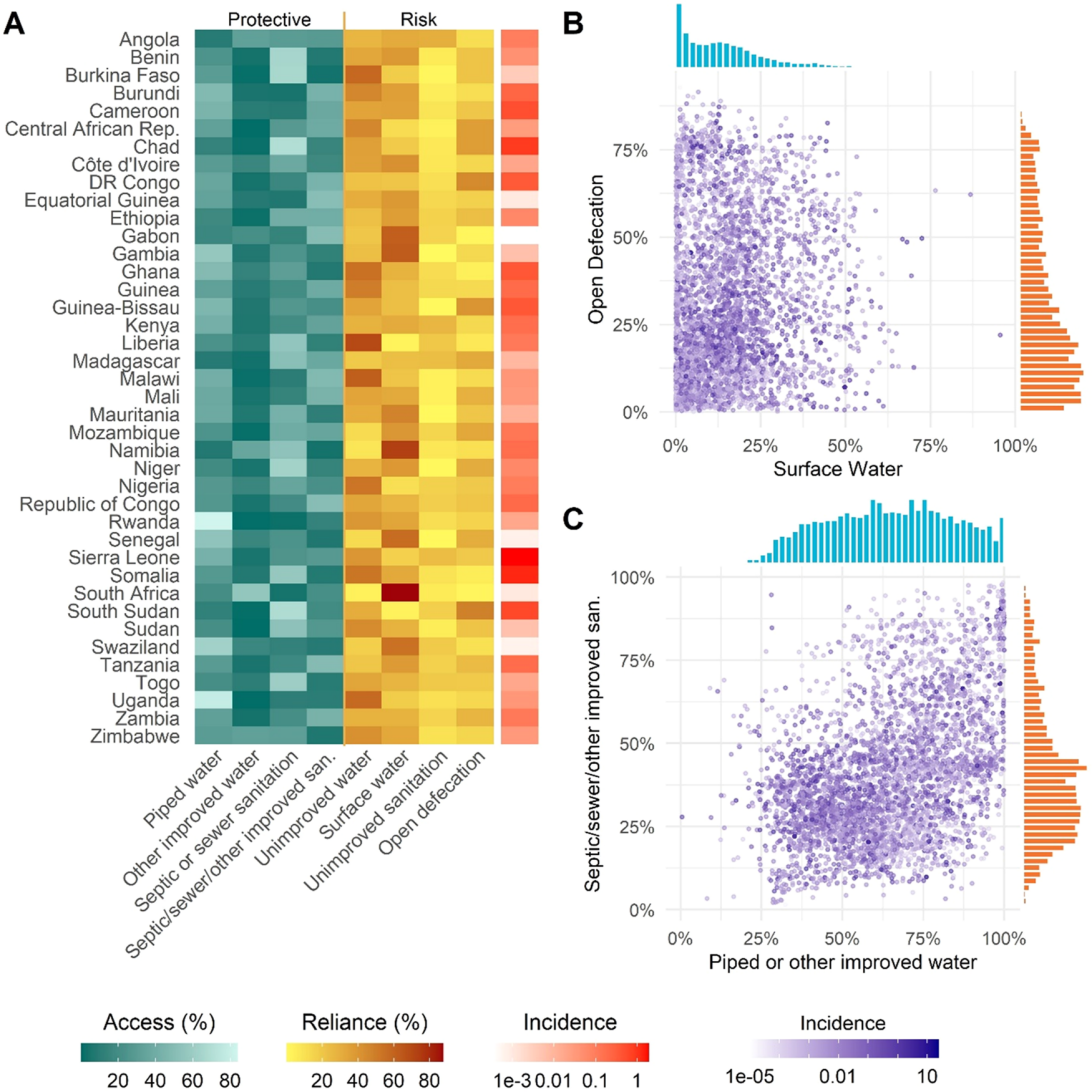
Water and sanitation measures and incidence rate of suspected cholera (2010–2016): (A) mean water and sanitation measures and log of mean annual incidence of suspected cholera per 1000 population by country; scatter plots with point color indicating mean annual incidence of suspected cholera cases per 1000 people as a function of (B) reliance on surface water and open defecation (extremes) by district; and (C) piped or other improved water and septic, sewer, or other improved sanitation by district. Univariate histograms of district-level measures shown along the axes of panels (B) and (C) are in blue and orange.

Country-level population-weighted mean water and sanitation measures varied across the study area (Figure 1 and Table S2).^10^ Of the four protective factors, access to improved water had the highest mean prevalence (mean: 68%, range: 34–92%) and access to septic or sewer sanitation had the lowest (10%, range: <1–55%). Among the risk factors, reliance on open defecation had the highest (32%, range: 2–70%) and reliance on surface water had the lowest prevalence (11%, <1–30%). The 2010–2016 mean annual incidence of suspected cholera at the national level ranged from 1.83 (95% CI 0.96–4.01) cases per 10 000 population (Sierra Leone) to 0.0003 (95% CI 0.0001–0.0005) cases per 10 000 population (Gabon).^11^

**Figure 2:**
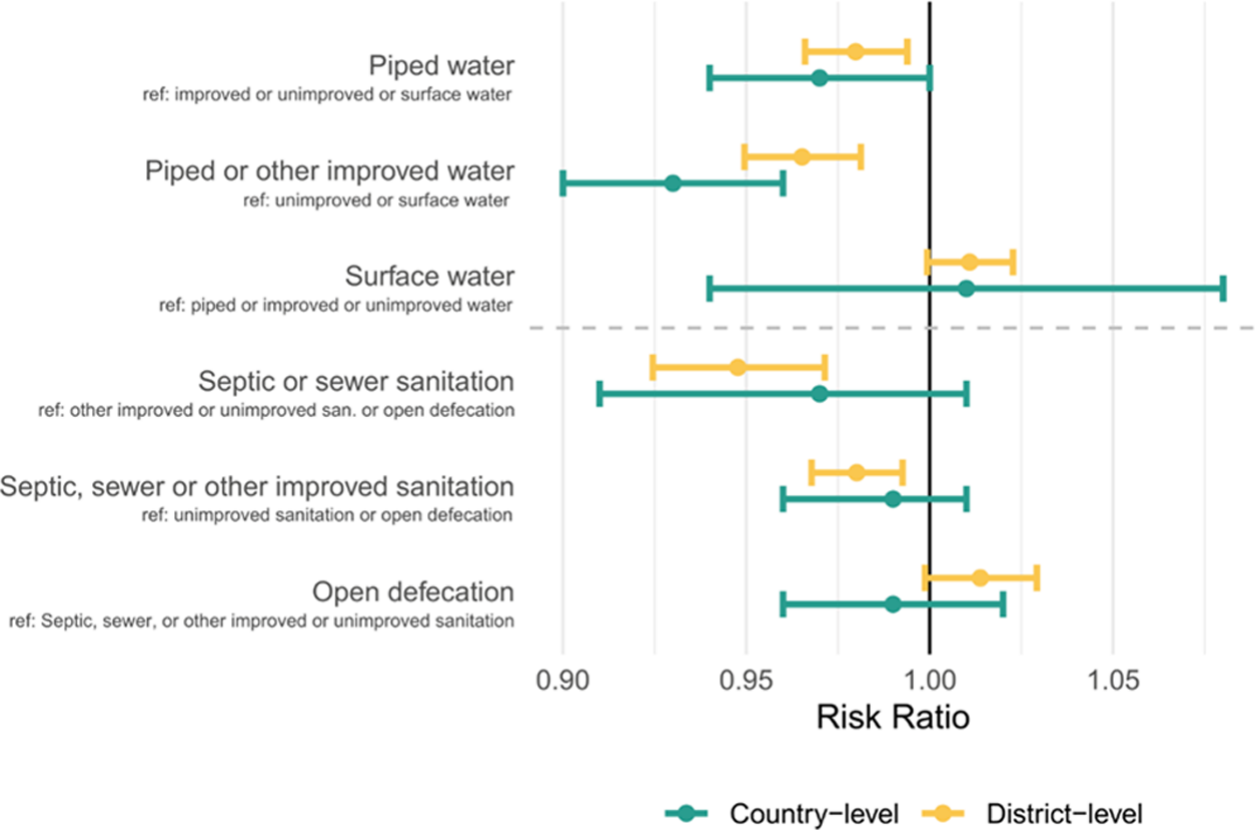
Risk ratio of water and sanitation measures from univariate models at country (Quasi-Poisson) and district scales (Poisson GEE). Reference groups (denoted “ref”) for each model are included to compare the risk ratios.

At the country level, we found that increases in access to piped or “other improved” water were associated with a significant decrease in mean annual cholera incidence in univariate analysis. A 1% increase in piped or “other improved” water access was associated with a 7.0% (95% CI 4.0–10.0) decrease in mean annual cholera incidence within the country. The remaining putative protective factors, access to piped water (3.0%, 95% CI 0.0–6.0); septic, sewer or “other improved” sanitation (1.0%, 95% CI −1.0–4.0); and septic or sewer sanitation (3.0%, 95% CI −1.0–9.0), had point estimates consistent with being protective though they were not significantly associated with mean annual cholera incidence (Figure 2). Among the putative risk factors, none achieved statistical significance at the 0.05 level. Effect estimates for reliance on surface water and open defecation were uncertain and confidence intervals spanned the null.

### District Level

Like the country level, the water and sanitation measures varied across the districts (Table S2). Of the four protective factors, access to improved water had the highest mean prevalence (mean: 64%, range: <1–100%) and access to septic or sewer sanitation had the lowest (8%, range: <1–95%). Among the risk factors, reliance on open defecation had the highest prevalence (32%, range: 2–93%) and reliance on surface water had the lowest (15%, 0–96%). The 2010–2016 mean annual incidence of suspected cholera at the district level ranged from 0 to 24 cases per 1000 population. Among the water and sanitation variables at the district level, piped or other improved water and septic, sewer, or other improved sanitation had the strongest correlation (Spearman rho = 0.42) (Figure 1C). Conversely, there was little correlation between reliance on surface water and open defecation, the two extremes of the service ladder (Spearman rho = 0.15) (Figure 1B).

When aggregating data to the district level, univariate results were qualitatively consistent with the country-level analyses. Increases in access to piped water, piped or “other improved” water, septic or sewer sanitation, and septic, sewer, or “other improved” sanitation were associated with a significant decrease in mean annual cholera incidence (Figure 2 and Table S3). For example, a 1% increase in access to piped or “other improved” water was associated with a 3.5% decrease (95% CI 1.9–5.1) in mean annual cholera incidence and a slightly larger reduction in incidence with septic or sewer sanitation (5.2% decrease, 95% CI 2.9–7.6). The proportion of the population using surface water was associated with an increase of mean annual incidence of 1.1% (95% CI −0.1–2.3%) and open defecation was associated with an increase of mean annual incidence of 1.4% (95% CI −0.1–2.9%), though neither was statistically significant (Figure 2 and Table S3).

**Figure 3:**
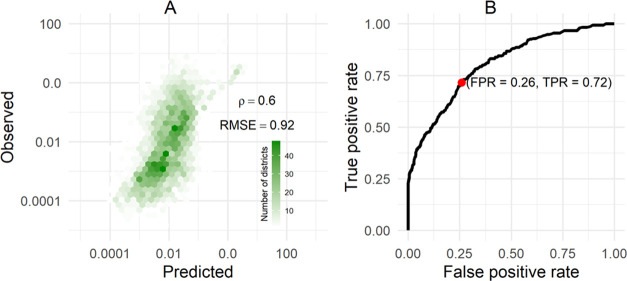
Summary of random forest models’ performance. Panel A illustrates the observed mean annual incidence versus the predicted values in cross-validation in the heatmap with each hexagon showing count of points. The root-mean-square error (RMSE) between the observed and model-predicted estimates and the correlation (\rho) between the two are noted in the panel. Panel B illustrates the cross-validated receiver operator characteristic curve from the random forest classification model used to identify high incidence areas. Youden cutoff point (jointly maximized sensitivity and specificity) shown as a red dot in panel B.

To better understand the potential predictive value of multiple water and sanitation measures combined for identifying high risk cholera areas, we used random forest regression models to predict mean annual cholera incidence and classification models to identify high incidence area districts. The cvRMSE of the random forest regression model was 0.92 (95% CI 0.90–0.94). With all six water and sanitation measures, the random forest model was able to explain 37% of the observed variability in mean annual cholera incidence in districts. The cross-validation predictions were weakly correlated with the true incidence (Spearman rho = 0.60) (Figure 3). In the full data model, the most influential predictors of mean annual incidence tended to be at the extreme ends of the water and sanitation hierarchy; open defecation followed by septic or sewer sanitation; piped water, surface water, and piped or “other improved” water; and septic, sewer, or “other improved” sanitation (Figure S1A). Similar qualitative results were found with the GBM model (see the Supporting Information), though the model did not fit as well (leave-one-district-out cvRMSE 1.06 95% CI 1.04–1.08), and piped water and septic or sewer sanitation were among the top three important variables in both models (Figure S1).

The random forest high incidence area classification model demonstrated moderate performance with a cvAUC of 0.81 (95% CI 0.78–0.83, Table S4). At the point that maximized model sensitivity and specificity, it incorrectly classified 28.5% of true high incidence areas (1-sensitivity) and falsely classified 26.0% of districts as high incidence areas (1-specificity). While the positive predictive value of these classification models varied across cutoff thresholds (range 7.6–98.6%), the negative predictive value remained high (range 92.5–100.0%, Table S5). This high negative predictive value suggests that even in the absence of reliable cholera incidence data, water and sanitation variables, ideally in conjunction with other factors, might be useful in screening out areas that are unlikely to be at risk for having high cholera incidence. The most influential water and sanitation measure in high incidence area classification models was surface water followed by septic or sewer sanitation, open defecation, piped water, piped or “other improved” water, and septic, sewer, or “other improved” sanitation (Figure S2A).

We found similar qualitative results using the GBM model (cvAUC 0.73, 95% CI 0.70–0.76; Table S4). When we focused only on the 25 countries that had at least one high cholera incidence area, we found that performances of the models were reduced (cvAUC 0.71 (95% CI 0.68–0.74) for random forest and 0.66 (95% CI 0.62–0.69) for the GBM model; Figure S3 and Table S4).

## Discussion

We investigated the relationship between water and sanitation access and suspected cholera incidence at both national and district levels across sub-Saharan Africa. While the direction of the association between individual water and sanitation measures and cholera generally aligned with prevailing evidence and beliefs, the size and significance of these associations varied across models and geographic scale of analysis. When combining all measures together, our classification models, while far from perfect, demonstrated moderate performance in discriminating high cholera incidence areas from areas without high incidence and may be especially useful in excluding potential high cholera incidence areas. As most of the evidence on water and sanitation and cholera come from small-scale highly local interventions, often in response to outbreaks, with outcomes measured over a short timeframe, our results help fill the evidentiary gap on the associations between multiannual cholera incidence rates and access to water and sanitation infrastructure in sub-Saharan Africa over the same period. They highlight some of the complexities of using these water and sanitation metrics in identifying priority targets for cholera control but suggest that there is likely value in including these in risk assessment.

Our results can be useful in discriminating between high and low cholera risk areas. Particularly, the water and sanitation measures that came across to be important in the regression and classification model are expected to contain useful information on cholera risk. When prioritizing cholera prevention interventions, the existing coverage of these measures in the target area can be used as decision-making criteria. However, aggregated coverage data should be used with caution as those may not reflect the vulnerability of the most susceptible group.

The variable association of water and sanitation estimates and cholera incidence between country- and district-level analyses indicates the importance of the spatial scale of analysis. Water and sanitation access can significantly vary within countries, within and between subnational units, and particularly between urban and rural areas,^20,21^ and while interventions like piped water might be implemented in whole jurisdictions, others like installation of sanitation facilities may occur on smaller scales. It is possible that more accurate and finer resolution data may have better predictive value for high cholera incidence areas, but these data are difficult to collect and rarely available when national cholera control planning is underway.

Beyond the general limitations of population-level analyses, the modeled gridded estimates of water and sanitation access and cholera incidence are imperfect given the sparse data in both data sets. Measures of access or reliance on different water and sanitation measures do not reflect behavior at the individual or population level, which directly influences disease risk; access is not a measure of usage and when used in analyses circumvents a step in the casual pathway leading to biased associations in either direction.^22^ Behavior data across geographies and at such large scales, however, is yet unavailable. Our water and sanitation service classifications were focused on facility types, which is generally what has been measured over the past decade through large representative surveys, whereas the new JMP service ladder included additional criteria to distinguish service levels. We included a supplementary table to elucidate the differences between these two views of water and sanitation (Table S6). Further, we used a machine learning approach to assess the association between water and sanitation and cholera without pre-specifying the functional form of the relationships and importance of different variables. Machine learning approaches have been used in the water, sanitation, and health sphere in the recent past for similar purposes,^23,24^ but their outputs can be difficult to translate into practical tools. The cholera incidence estimates were based primarily on data of reported suspected, not laboratory confirmed, cases. Among suspected cases, there are almost certainly those with diarrhea caused by other pathogens than *V. cholerae* O1/O139;^25^ similarly, under-reporting can lower the actual incidence. Additionally, these estimates represented an average over time, which may have attenuated the apparent associations between measures. Previous work has suggested that incomplete and erroneous reporting in water quality can produce misleading results,^26^ which would then propagate to modeled estimates.

Our results are based on suspected cholera incidence estimates from 2010 to 2016; as newer and perhaps better data become available, updates to these analyses should be performed. Additionally, conflict and disasters can lead to a significant reduction in access to water and sanitation infrastructure, which in turn can elevate the risk of cholera in areas that previously had a low incidence of the disease. Finally, our analysis measures cholera risk only through mean annual incidence, but metrics like outbreak size and frequency^27^ may provide different insights into the relationship between water and sanitation access and cholera transmission.

Our analysis highlights the association of water and sanitation measures in estimating cholera incidence and the potential power of district-level estimates of piped and other improved water and sanitation to help identify areas with cholera risk. Targeting oral cholera vaccination—a strategy to reduce near-term cholera risk^9^—according to water and sanitation measures may not outperform the vaccine impact of targeting based on historical cholera incidence.^28^ However, our analysis demonstrates that monitoring data on access to water and sanitation should remain an important component in cholera control and factor into priority setting for National Cholera Plans (NCPs). To that end, Global Task Force on Cholera Control (GTFCC) guidance for prioritizing areas for multisectoral interventions focuses primarily on measures of historical cholera suspected incidence, but a recent update^29^ proposes the use of water and sanitation access as risk factors for intervention prioritization, particularly when areas have known gaps in historical surveillance data or no recent cholera outbreaks. The absence of high-quality water and sanitation access estimates at the operational scales for cholera control poses a great challenge to their systematic integration into prioritization activities. Future work to collect more precise and health-relevant metrics on water and sanitation (e.g., water quality tests, service quality indicators and other metrics following revised JMP standards on safely managed services,^30^ data on water and sanitation related behaviors, and data on confirmed, rather than suspected cholera), can help refine our understanding of these important relationships.

## Supporting Information Available

Tables and figures showing country-level data summary; country- and district-level water and sanitation mean percent access/reliance; univariate Poisson regression results; multivariate random forest and GBM cross-validation evaluation matric; random forest classification model performance metrics for cutoff thresholds; multivariate GBM process; variable importance for regression and classification models; high cholera incidence analysis, AUC, and variable importance; and the comparison between water sanitation indicators and JMP service ladder (PDF)
