# Supplementary material for: Water, Sanitation, and Cholera in Sub-Saharan Africa
Source: Environ Sci Technol. 2023 Jul 6;57(28):10185–92. doi: 10.1021/acs.est.3c01317 (PMC10357557; doi:10.1021/acs.est.3c01317)
Supplement: Supplementary file 1 — es3c01317_si_001.pdf [file es3c01317_si_001.pdf]

# Water, Sanitation and Cholera in sub-Saharan Africa

Mustafa Sikder,[1] Aniruddha Deshpande,[2] Sonia T. Hegde,[3] Espoir Bwenge Malembaka,[3, 4] Karin Gallandat,[5] Robert C. Reiner,[6] Justin Lessler,[3] Elizabeth C. Lee,[3] Andrew S. Azman*[3]

[1] Department of International Health, Johns Hopkins Bloomberg School of Public Health, Johns Hopkins University, Baltimore, Maryland 21205, United States
[2] Institute for Health Metrics and Evaluation, University of Washington, Seattle, Washington 98105, United States
[3] Department of Epidemiology, Johns Hopkins Bloomberg School of Public Health, Johns Hopkins University, Baltimore, Maryland 21205, United States
[4] Center for Tropical Diseases and Global Health (CTDGH), Université Catholique de Bukavu (UCB), Bukavu, the Democratic Republic of Congo.
[5] Environmental Health Group, Department for Disease Control, Faculty of Infectious and Tropical Diseases, London School of Hygiene and Tropical Medicine, London WC1E 7HT, United Kingdom
[6] Department of Health Metrics Sciences, School of Medicine, University of Washington, Seattle, Washington 98105, United States
[7] Geneva Centre for Emerging Viral Diseases, Geneva University Hospitals, Geneva, Switzerland
[8] Division of Tropical and Humanitarian Medicine, Geneva University Hospitals, Geneva, Switzerland

Number of pages: 1-10
Figures: S1-S3
Tables: S1-S6

Table of contents

Figures:

Figure S1: Incidence random forest (RF) and gradient boosting machine (GBM) variable importance ....6
Figure S2: Classification random forest (RF) and gradient boosting machine (GBM) variable importance ....7
Figure S3: Random forest classification for high cholera incidence district; (A) cross-validation model area under the receiver operator characteristic curve plot; (B) conditional permutation variable importance plot ....7

Tables:

Table S1. Access/reliance to water and sanitation measures in percentage and incidence of cholera in 1,000 by country 2
Table S2. Mean percentage of access or reliance on water and sanitation measures at the country and district level .....3
Table S3. Risk ratio of cholera incidence for water and sanitation measures at the country and district level....4
Table S4. Leave-one-district out and leave-one-country out cross-validation results ....4
Table S5. Random forest classification model performance metrics for different cutoff thresholds ....5
Table S6. Water and sanitation indicators and Joint Monitoring Program service ladder....8

# Supporting Information

## Country level Data

The population weighted water and sanitation access/reliance percentage and incidence of cholera per 1,000 by country are presented in Table S1.

Table S1. Access/reliance to water and sanitation measures in percentage and incidence of cholera in 1,000 by country

| Country | Piped Water | Other Improved Water | Septic or sewer sanitation | Other Improved Sanitation | Unimproved water | Surface Water | Unimproved Sanitation | Open Defecation | Incidence/1,000 |
|---|---|---|---|---|---|---|---|---|---|
| Angola | 32.0 | 26.6 | 32.6 | 9.5 | 11.5 | 29.9 | 28.4 | 29.5 | 0.09904 |
| Burundi | 36.4 | 46.4 | 5.0 | 47.6 | 11.4 | 5.8 | 43.1 | 4.3 | 0.24680 |
| Benin | 39.1 | 33.2 | 3.6 | 23.9 | 21.6 | 6.1 | 7.7 | 64.8 | 0.04665 |
| Burkina Faso | 18.0 | 58.4 | 1.3 | 29.6 | 21.7 | 1.9 | 3.3 | 65.8 | 0.00358 |
| Central African Rep. | 12.1 | 46.4 | 0.4 | 32.3 | 37.6 | 4.0 | 39.1 | 28.2 | 0.02886 |
| Côte d'Ivoire | 42.6 | 35.2 | 15.8 | 29.2 | 14.3 | 7.8 | 17.2 | 37.7 | 0.01891 |
| Cameroon | 35.7 | 34.6 | 9.7 | 44.6 | 20.7 | 9.0 | 37.2 | 8.5 | 0.50913 |
| DR Congo | 21.2 | 22.3 | 3.9 | 35.9 | 45.3 | 11.2 | 43.9 | 16.3 | 0.36399 |
| Republic of Congo | 27.1 | 34.2 | 4.8 | 26.2 | 21.3 | 17.4 | 50.3 | 18.8 | 0.20317 |
| Ethiopia | 37.5 | 23.2 | 1.9 | 17.2 | 23.4 | 16.0 | 40.6 | 40.3 | 0.06755 |
| Gabon | 61.9 | 20.7 | 20.9 | 16.0 | 1.8 | 15.6 | 49.3 | 13.8 | 0.00033 |
| Ghana | 28.3 | 54.9 | 13.3 | 48.3 | 4.7 | 12.1 | 7.6 | 30.9 | 0.40156 |
| Guinea | 23.9 | 49.8 | 9.5 | 32.3 | 13.0 | 13.3 | 38.6 | 19.6 | 0.20086 |
| Gambia | 63.0 | 23.4 | 7.3 | 56.2 | 13.4 | 0.2 | 12.3 | 24.1 | 0.00612 |
| Guinea-Bissau | 23.8 | 34.7 | 7.0 | 46.0 | 41.1 | 0.4 | 20.8 | 26.1 | 0.39827 |
| Equatorial Guinea | 39.0 | 30.8 | 8.3 | 33.8 | 16.3 | 13.8 | 50.9 | 7.0 | 0.00100 |
| Kenya | 31.5 | 28.5 | 6.2 | 41.5 | 13.4 | 26.6 | 33.4 | 18.9 | 0.17645 |
| Liberia | 2.9 | 69.8 | 9.8 | 22.8 | 7.5 | 19.8 | 12.5 | 54.9 | 0.11201 |
| Madagascar | 23.9 | 19.1 | 3.2 | 8.6 | 32.7 | 24.3 | 36.8 | 51.4 | 0.00895 |
| Mali | 32.6 | 34.4 | 4.1 | 37.8 | 29.1 | 3.9 | 44.1 | 14.0 | 0.03260 |
| Mozambique | 39.9 | 12.5 | 1.3 | 24.2 | 33.0 | 14.6 | 35.1 | 39.4 | 0.12538 |
| Mauritania | 48.1 | 31.0 | 7.1 | 38.4 | 20.4 | 0.5 | 11.6 | 42.9 | 0.01136 |
| Malawi | 21.3 | 62.1 | 1.5 | 40.9 | 12.6 | 4.1 | 47.7 | 9.9 | 0.03755 |
| Namibia | 73.2 | 8.5 | 35.9 | 7.6 | 10.5 | 7.7 | 3.4 | 53.1 | 0.17558 |
| Niger | 39.6 | 28.0 | 4.7 | 18.4 | 31.4 | 1.0 | 14.1 | 62.8 | 0.06494 |

| Country | Piped Water | Other Improved Water | Septic or sewer sanitation | Other Improved Sanitation | Unimproved water | Surface Water | Unimproved Sanitation | Open Defecation | Incidence/1,000 |
|---|---|---|---|---|---|---|---|---|---|
| Nigeria | 11.1 | 53.1 | 15.8 | 27.1 | 19.4 | 16.4 | 22.0 | 35.2 | 0.10247 |
| Rwanda | 33.9 | 39.8 | 0.6 | 84.1 | 15.1 | 11.2 | 13.4 | 1.9 | 0.01835 |
| Sudan | 28.3 | 44.7 | 0.1 | 20.3 | 21.5 | 5.6 | 27.0 | 52.7 | 0.00598 |
| Senegal | 56.8 | 12.3 | 15.8 | 53.7 | 30.5 | 0.4 | 6.9 | 23.6 | 0.00072 |
| Sierra Leone | 16.1 | 40.3 | 4.8 | 42.7 | 19.4 | 24.2 | 28.0 | 24.5 | 1.89451 |
| Somalia | 32.3 | 52.8 | 7.6 | 27.5 | 5.3 | 9.6 | 7.8 | 57.1 | 1.27452 |
| South Sudan | 3.2 | 30.9 | 0.7 | 12.1 | 48.7 | 17.2 | 18.9 | 68.4 | 0.61066 |
| Swaziland | 54.3 | 16.9 | 16.2 | 62.3 | 10.1 | 18.7 | 8.7 | 12.8 | 0.00065 |
| Chad | 18.9 | 36.3 | 1.6 | 13.5 | 37.5 | 7.2 | 15.4 | 69.5 | 0.86626 |
| Togo | 27.6 | 33.9 | 10.4 | 20.3 | 18.9 | 19.7 | 9.2 | 60.1 | 0.01796 |
| Tanzania | 38.2 | 20.4 | 4.9 | 29.1 | 25.0 | 16.4 | 49.0 | 17.0 | 0.18485 |
| Uganda | 18.0 | 57.8 | 0.2 | 78.7 | 13.6 | 10.6 | 10.2 | 10.9 | 0.03673 |
| South Africa | 87.6 | 3.9 | 55.4 | 19.8 | 2.3 | 6.2 | 20.8 | 4.0 | 0.00103 |
| Zambia | 28.4 | 28.9 | 3.7 | 32.2 | 27.4 | 15.4 | 43.6 | 20.6 | 0.11792 |
| Zimbabwe | 32.9 | 42.6 | 32.3 | 29.4 | 14.6 | 9.9 | 6.9 | 31.4 | 0.03265 |

## Country and district level access/reliance summary

The country and district level aggregated mean percent of access or reliance on water and sanitation measures from previously published work are present in Table S2.[1]

Table S2. Mean percentage of access or reliance on water and sanitation measures at the country and district level

| | **Mean percent of access/reliance (min-max)** | |
|---|---|---|
| | Country level | District level |
| Improved water | 68.1 (34.1-91.5) | 64.2 (0.4-99.9) |
| Piped water | 33.6 (2.90- 87.6) | 26.2 (<0.1-99.9) |
| Improved sanitation | 42.8 (11.8- 84.7) | 40.7 (1.6-98.3) |
| Septic or sewer sanitation | 9.73 (<0.1- 55.4) | 8.2 (<0.1-95.1) |
| Unimproved water | 20.5 (1.8- 48.7) | 20.7 (0-90.9) |
| Surface water | 11.4 (0.2- 29.9) | 15.2 (0-95.5) |
| Unimproved sanitation | 25.4 (3.3- 50.9) | 28.2 (<0.1-87.1) |

| | | |
|---|---|---|
| Open defecation | 31.8 (1.9- 69.5) | 31.2 (0.1-92.9) |

## Univariate analysis

Country and district level univariate analysis with water and sanitation measures as predictor and incidence of cholera as outcome variable are presented in Table S3. We use Quasi-Poisson regression with water and sanitation measures as linear predictors of cholera incidence and total population of the country as an offset term. For district level analysis, we used Poisson generalized estimating equations (GEE) with country as cluster variable and district population as an offset term.

Table S3. Risk ratio of cholera incidence for water and sanitation measures at the country and district level

| | **Risk ratio (95% confidence interval)** | |
|---|---|---|
| | Country level | Second-level |
| Piped water | 0.97 (0.94-1) | 0.98 (0.97-0.99) |
| Piped or other improved | 0.93 (0.9-0.96) | 0.97 (0.95-0.98) |
| Surface water | 1.01 (0.94-1.08) | 1.01 (1-1.02) |
| Septic or sewer sanitation | 0.97 (0.91-1.01) | 0.95 (0.92-0.97) |
| Piped or other improved sanitation | 0.99 (0.96-1.01) | 0.98 (0.97-0.99) |
| Open defecation | 0.99 (0.96-1.02) | 1.01 (1-1.03) |

## Multivariate analysis

We implemented cross-validation to evaluate model performance. In the leave-leave-one-district out process, we trained the model N= 4,146 times using all except one (N-1) data point and predicted for that data point. Additionally, we experimented with leave-one-country out cross-validation step (Table S4). In this process, we trained the model N= 40 times, each time leaving all data from one country out for evaluation. The sample in each training and evaluation varied according to the number of districts in each country. The length of training data varied from 3,355 to 4,133 and the test data from 13 to 791.

Table S4. Leave-one-district out and leave-one-country out cross-validation results

| Model type (evaluation metric) | Algorithm | Cross-validation (95% CI) | |
|---|---|---|---|
| | | Leave-one-district out | Leave-one-country out |
| Regression (cvRMSE) | Random forest | 0.92 (0.90-0.94) | 0.97 (0.94- 1.0) |
| | GBM | 1.06 (1.04-1.08) | 1.07 (1.04-1.12) |
| Classification (cvAUC) | Random forest | 0.81 (0.78-0.83) | 0.73 (0.73-0.76) |

| | GBM | 0.73 (0.70-0.76 | 0.51(0.48-0.54) |
|---|---|---|---|
| High incidence area classification (cvAUC) | Random forest | 0.71 (0.68-0.74) | 0.65 (0.61-0.69) |
| | GBM | 0.66 (0.62-0.69) | 0.65 (0.62-0.69) |

Note: cvRMSE= cross-validation root-mean-square error; cvAUC= cross-validation area under the curve; GBM= gradient boosting machine

## Multivariate random forest classification

Cutoff threshold, true positive rate, false positive rate, positive predictive value, negative predictive value of the random forest classification model using leave-one-district cross-validation (Table S5).

Table S5. Random forest classification model performance metrics for different cutoff thresholds

| Cutoff | True positive rate (sensitivity) | Specificit y | False positive rate | Positive predictive value | Negative predictive value | Youden |
|---|---|---|---|---|---|---|
| 0.00 | 1.000000 | 0.018243 | 0.981757 | 0.075810 | 1.000000 | 0.018243 |
| 0.01 | 0.993528 | 0.076883 | 0.923117 | 0.079761 | 0.993266 | 0.070410 |
| 0.02 | 0.983819 | 0.142299 | 0.857701 | 0.084562 | 0.990926 | 0.126117 |
| 0.03 | 0.967638 | 0.207454 | 0.792546 | 0.089521 | 0.987593 | 0.175091 |
| 0.04 | 0.954693 | 0.264790 | 0.735210 | 0.094673 | 0.986408 | 0.219483 |
| 0.05 | 0.948220 | 0.316393 | 0.683607 | 0.100480 | 0.986992 | 0.264613 |
| 0.06 | 0.935275 | 0.366432 | 0.633568 | 0.106250 | 0.985975 | 0.301707 |
| 0.07 | 0.925566 | 0.408913 | 0.591087 | 0.111981 | 0.985553 | 0.334480 |
| 0.08 | 0.899676 | 0.442794 | 0.557206 | 0.115066 | 0.982081 | 0.342470 |
| 0.09 | 0.886731 | 0.476414 | 0.523586 | 0.120018 | 0.981213 | 0.363145 |
| 0.10 | 0.870550 | 0.503258 | 0.496742 | 0.123678 | 0.979706 | 0.373808 |
| 0.11 | 0.857605 | 0.534793 | 0.465207 | 0.129268 | 0.979008 | 0.392398 |
| 0.12 | 0.844660 | 0.562679 | 0.437321 | 0.134605 | 0.978251 | 0.407339 |
| 0.13 | 0.831715 | 0.585874 | 0.414126 | 0.139220 | 0.977391 | 0.417590 |
| 0.14 | 0.825243 | 0.608027 | 0.391973 | 0.144969 | 0.977377 | 0.433270 |
| 0.15 | 0.802589 | 0.627834 | 0.372166 | 0.147971 | 0.975304 | 0.430423 |
| 0.16 | 0.792880 | 0.650508 | 0.349492 | 0.154477 | 0.975000 | 0.443388 |
| 0.17 | 0.783172 | 0.669012 | 0.330988 | 0.160053 | 0.974563 | 0.452184 |
| 0.18 | 0.760518 | 0.687516 | 0.312484 | 0.163877 | 0.972714 | 0.448034 |
| 0.19 | 0.747573 | 0.703935 | 0.296065 | 0.168983 | 0.971932 | 0.451508 |
| 0.20 | 0.734628 | 0.717488 | 0.282512 | 0.173150 | 0.971076 | 0.452115 |
| 0.21 | 0.715210 | 0.739640 | 0.260360 | 0.181148 | 0.969925 | 0.454851 |
| 0.22 | 0.699029 | 0.751889 | 0.248111 | 0.184932 | 0.968771 | 0.450919 |
| 0.23 | 0.673139 | 0.765963 | 0.234037 | 0.188065 | 0.966776 | 0.439102 |
| 0.24 | 0.647249 | 0.779255 | 0.220745 | 0.191022 | 0.964827 | 0.426504 |
| 0.25 | 0.627832 | 0.789940 | 0.210060 | 0.194000 | 0.963446 | 0.417772 |
| 0.26 | 0.611650 | 0.798801 | 0.201199 | 0.196670 | 0.962323 | 0.410452 |
| 0.27 | 0.601942 | 0.811050 | 0.188950 | 0.204171 | 0.961978 | 0.412992 |
| 0.28 | 0.582524 | 0.820172 | 0.179828 | 0.206897 | 0.960623 | 0.402696 |
| 0.29 | 0.572816 | 0.829554 | 0.170446 | 0.212996 | 0.960181 | 0.402370 |
| 0.30 | 0.566343 | 0.843888 | 0.156112 | 0.226098 | 0.960261 | 0.410231 |
| 0.31 | 0.550162 | 0.853531 | 0.146469 | 0.232240 | 0.959285 | 0.403693 |
| 0.32 | 0.527508 | 0.862653 | 0.137347 | 0.236232 | 0.957755 | 0.390161 |
| 0.33 | 0.517799 | 0.871254 | 0.128746 | 0.244648 | 0.957331 | 0.389053 |
| 0.34 | 0.514563 | 0.878290 | 0.121710 | 0.253994 | 0.957386 | 0.392853 |
| 0.35 | 0.495146 | 0.886370 | 0.113630 | 0.259762 | 0.956143 | 0.381515 |
| 0.36 | 0.478964 | 0.895491 | 0.104509 | 0.269581 | 0.955240 | 0.374456 |
| 0.37 | 0.475728 | 0.902007 | 0.097993 | 0.281071 | 0.955286 | 0.377735 |
| 0.38 | 0.472492 | 0.909825 | 0.090175 | 0.296748 | 0.955391 | 0.382317 |
| 0.39 | 0.466019 | 0.916602 | 0.083398 | 0.310345 | 0.955187 | 0.382621 |

| Cutoff | True positive rate (sensitivity) | Specificity | False positive rate | Positive predictive value | Negative predictive value | Youden |
|---|---|---|---|---|---|---|
| 0.40 | 0.456311 | 0.921293 | 0.078707 | 0.318284 | 0.954631 | 0.377603 |
| 0.41 | 0.449838 | 0.924681 | 0.075319 | 0.324766 | 0.954276 | 0.374519 |
| 0.42 | 0.440129 | 0.930675 | 0.069325 | 0.338308 | 0.953793 | 0.370804 |
| 0.43 | 0.427184 | 0.936669 | 0.063331 | 0.352000 | 0.953063 | 0.363854 |
| 0.44 | 0.411003 | 0.942142 | 0.057858 | 0.363897 | 0.952067 | 0.353146 |
| 0.45 | 0.404531 | 0.947355 | 0.052645 | 0.382263 | 0.951820 | 0.351885 |
| 0.46 | 0.398058 | 0.952567 | 0.047433 | 0.403279 | 0.951575 | 0.350625 |
| 0.47 | 0.394822 | 0.956737 | 0.043263 | 0.423611 | 0.951529 | 0.351559 |
| 0.48 | 0.391586 | 0.959083 | 0.040917 | 0.435252 | 0.951396 | 0.350668 |
| 0.49 | 0.381877 | 0.962471 | 0.037529 | 0.450382 | 0.950824 | 0.344348 |
| 0.50 | 0.355987 | 0.965338 | 0.034662 | 0.452675 | 0.949014 | 0.321325 |
| 0.51 | 0.352751 | 0.968986 | 0.031014 | 0.478070 | 0.948954 | 0.321737 |
| 0.52 | 0.343042 | 0.970550 | 0.029450 | 0.484018 | 0.948307 | 0.313592 |
| 0.53 | 0.320388 | 0.973156 | 0.026844 | 0.490099 | 0.946755 | 0.293544 |
| 0.54 | 0.317152 | 0.977326 | 0.022674 | 0.529730 | 0.946731 | 0.294478 |
| 0.55 | 0.310680 | 0.979411 | 0.020589 | 0.548571 | 0.946361 | 0.290091 |
| 0.56 | 0.300971 | 0.981235 | 0.018765 | 0.563636 | 0.945742 | 0.282206 |
| 0.57 | 0.291262 | 0.983320 | 0.016680 | 0.584416 | 0.945140 | 0.274582 |
| 0.58 | 0.291262 | 0.983581 | 0.016419 | 0.588235 | 0.945154 | 0.274843 |
| 0.59 | 0.291262 | 0.985145 | 0.014855 | 0.612245 | 0.945236 | 0.276407 |
| 0.60 | 0.291262 | 0.987490 | 0.012510 | 0.652174 | 0.945359 | 0.278752 |
| 0.61 | 0.281553 | 0.990096 | 0.009904 | 0.696000 | 0.944790 | 0.271650 |
| 0.62 | 0.281553 | 0.991660 | 0.008340 | 0.731092 | 0.944872 | 0.273214 |
| 0.63 | 0.281553 | 0.992963 | 0.007037 | 0.763158 | 0.944940 | 0.274517 |
| 0.64 | 0.275081 | 0.993224 | 0.006776 | 0.765766 | 0.944486 | 0.268305 |
| 0.65 | 0.275081 | 0.993484 | 0.006516 | 0.772727 | 0.944500 | 0.268565 |
| 0.66 | 0.271845 | 0.994527 | 0.005473 | 0.800000 | 0.944321 | 0.266372 |
| 0.67 | 0.268608 | 0.994527 | 0.005473 | 0.798077 | 0.944087 | 0.263135 |
| 0.68 | 0.265372 | 0.994527 | 0.005473 | 0.796117 | 0.943854 | 0.259899 |
| 0.69 | 0.255663 | 0.995048 | 0.004952 | 0.806122 | 0.943182 | 0.250712 |
| 0.70 | 0.252427 | 0.995048 | 0.004952 | 0.804124 | 0.942949 | 0.247475 |
| 0.71 | 0.252427 | 0.995830 | 0.004170 | 0.829787 | 0.942991 | 0.248257 |
| 0.72 | 0.249191 | 0.996091 | 0.003909 | 0.836957 | 0.942773 | 0.245282 |
| 0.73 | 0.249191 | 0.996351 | 0.003649 | 0.846154 | 0.942787 | 0.245542 |
| 0.74 | 0.249191 | 0.996873 | 0.003127 | 0.865169 | 0.942815 | 0.246063 |
| 0.75 | 0.249191 | 0.996873 | 0.003127 | 0.865169 | 0.942815 | 0.246063 |
| 0.76 | 0.245955 | 0.997133 | 0.002867 | 0.873563 | 0.942597 | 0.243088 |
| 0.77 | 0.239482 | 0.997654 | 0.002346 | 0.891566 | 0.942161 | 0.237137 |
| 0.78 | 0.239482 | 0.997915 | 0.002085 | 0.902439 | 0.942175 | 0.237397 |
| 0.79 | 0.239482 | 0.997915 | 0.002085 | 0.902439 | 0.942175 | 0.237397 |
| 0.80 | 0.239482 | 0.998176 | 0.001824 | 0.913580 | 0.942189 | 0.237658 |
| 0.81 | 0.239482 | 0.998176 | 0.001824 | 0.913580 | 0.942189 | 0.237658 |
| 0.82 | 0.239482 | 0.998436 | 0.001564 | 0.925000 | 0.942204 | 0.237918 |
| 0.83 | 0.239482 | 0.998697 | 0.001303 | 0.936709 | 0.942218 | 0.238179 |
| 0.84 | 0.236246 | 0.998958 | 0.001042 | 0.948052 | 0.942000 | 0.235203 |
| 0.85 | 0.236246 | 0.998958 | 0.001042 | 0.948052 | 0.942000 | 0.235203 |
| 0.86 | 0.233010 | 0.999479 | 0.000521 | 0.972973 | 0.941798 | 0.232488 |
| 0.87 | 0.233010 | 0.999479 | 0.000521 | 0.972973 | 0.941798 | 0.232488 |
| 0.88 | 0.233010 | 0.999479 | 0.000521 | 0.972973 | 0.941798 | 0.232488 |
| 0.89 | 0.233010 | 0.999739 | 0.000261 | 0.986301 | 0.941812 | 0.232749 |
| 0.90 | 0.233010 | 0.999739 | 0.000261 | 0.986301 | 0.941812 | 0.232749 |
| 0.91 | 0.229773 | 1.000000 | 0.000000 | 1.000000 | 0.941595 | 0.229773 |
| 0.92 | 0.229773 | 1.000000 | 0.000000 | 1.000000 | 0.941595 | 0.229773 |
| 0.93 | 0.229773 | 1.000000 | 0.000000 | 1.000000 | 0.941595 | 0.229773 |
| 0.94 | 0.229773 | 1.000000 | 0.000000 | 1.000000 | 0.941595 | 0.229773 |
| 0.95 | 0.229773 | 1.000000 | 0.000000 | 1.000000 | 0.941595 | 0.229773 |
| 0.96 | 0.229773 | 1.000000 | 0.000000 | 1.000000 | 0.941595 | 0.229773 |
| 0.97 | 0.223301 | 1.000000 | 0.000000 | 1.000000 | 0.941133 | 0.223301 |
| 0.98 | 0.223301 | 1.000000 | 0.000000 | 1.000000 | 0.941133 | 0.223301 |

| Cutoff | True positive rate (sensitivity) | Specificit y | False positive rate | Positive predictive value | Negative predictive value | Youden |
|---|---|---|---|---|---|---|
| 0.99 | 0.213592 | 1.000000 | 0.000000 | 1.000000 | 0.940441 | 0.213592 |
| 1.00 | 0.000000 | 1.000000 | 0.000000 | NA | 0.925470 | 0.000000 |

## Multivariate GBM:

Following the same approach as reported in Results, we applied gradient boosting machine (GBM) regression models on the cholera incidence data and classification models to predict high cholera incidence areas. The cvRMSE of the GBM cholera incidence model was 1.07 (95% CI 1.04-1.12) and the full data model used septic or sewer sanitation, piped or other improved water, and piped water as the top three important variable to predict cholera incidences (Figure S1 B). The cvAUC of the GBM high cholera incidence area model was 0.66 (95% CI 0.62-0.69) and the full data model used piped water, piped or other improved water, surface water as the top three important variables to predict high cholera incidence areas (Figure S2 B).

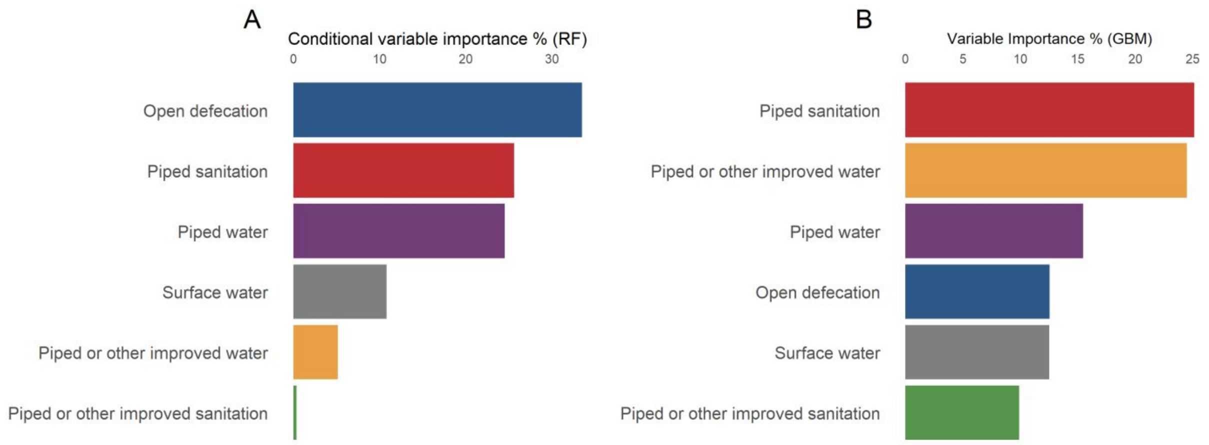


Figure S1: Incidence random forest (RF) and gradient boosting machine (GBM) variable importance

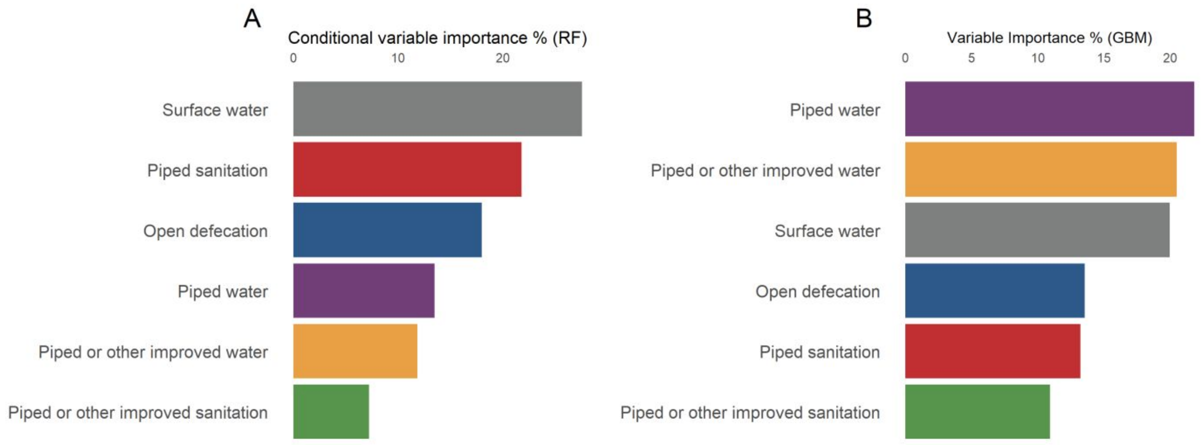


Figure S2: Classification random forest (RF) and gradient boosting machine (GBM) variable importance

## High cholera incidence districts

To understand the water and sanitation and cholera relationship in high incidence countries, we focused only on the 25 countries that had at least one high cholera incidence districts. We found that performances of the models were reduced (cvAUC 0.71 (95% CI 0.68-0.74) for random forest and 0.66 (95% CI 0.62-0.69) for the GBM model; Figure S3, Table S3).

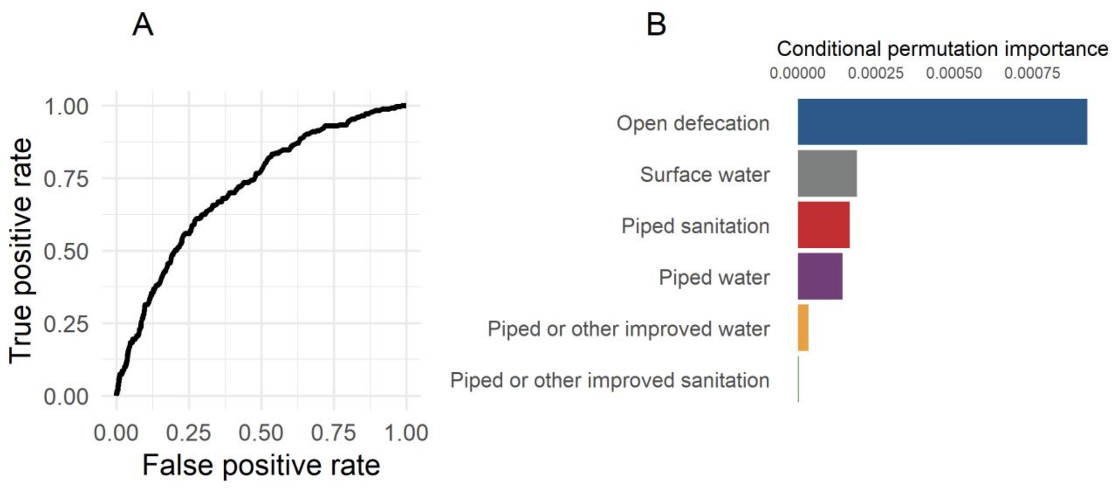


Figure S3: Random forest classification for high cholera incidence district; (A) cross-validation model area under the receiver operator characteristic curve plot; (B) conditional permutation variable importance plot

## Water sanitation indicators and JMP service ladder

We used previously published water and sanitation indicators[1] which focused on access by facility type classification. The current Joint Monitoring Program (JMP) service ladders are built on the previous improved/unimproved facility type classification but includes additional criteria relating to service levels. Table S6 reports the comparability between the two classifications.

Table S6. Water and sanitation indicators and Joint Monitoring Program service ladder

| | **Access/reliance indicator** | **JMP service ladder** |
|---|---|---|
| **Water** | **Piped water** (on or off premises)<br>+<br>**Other improved water** (on or off premises). | **Safely managed**: Drinking water from an improved water source that is accessible on premises, available when needed and free from fecal and priority chemical contamination |
| | | **Basic**: Drinking water from an improved source, provided collection time is not more than 30 minutes for a roundtrip including queuing |
| | | **Limited**: Drinking water from an improved source for which collection time exceeds 30 minutes for a roundtrip including queuing |
| | **Unimproved water** | **Unimproved**: Drinking water from an unprotected dug well or unprotected spring |

| | | |
|---|---|---|
| | **Surface water** | **Surface water**: Drinking water directly from a river, dam, lake, pond, stream, canal or irrigation canal |
| **Sanitation** | **Septic or sewer sanitation** + **Other improved sanitation** | **Safely managed**: Use of improved facilities that are not shared with other households and where excreta are safely disposed of in situ or removed and treated offsite |
| | | **Basic**: Use of improved facilities which are not shared with other households |
| | | **Limited**: Use of improved facilities shared between two or more households |
| | **Unimproved sanitation** (unimproved latrines, bucket, hanging toilet) | **Unimproved**: Use of pit latrines without a slab or platform, hanging latrines or bucket latrines |
| | **Open defecation** | **Open defecation**: Disposal of human faeces in fields, forests, bushes, open bodies of water, beaches and other open spaces or with solid waste |

## References

(1) Deshpande, A.; Miller-Petrie, M. K.; Lindstedt, P. A.; Baumann, M. M.; Johnson, K. B.; Blacker, B. F.; Abbastabar, H.; Abd-Allah, F.; Abdelalim, A.; Abdollahpour, I.; Abegaz, K. H.; Abejie, A. N.; Abreu, L. G.; Abrigo, M. R. M.; Abualhasan, A.; Accrombessi, M. M. K.; Adamu, A. A.; Adebayo, O. M.; Adedeji, I. A.; Adedoyin, R. A.; Adekanmbi, V.; Adetokunboh, O. O.; Adhikari, T. B.; Afarideh, M.; Agudelo-Botero, M.; Ahmadi, M.; Ahmadi, K.; Ahmed, M. B.; Ahmed, A. E.; Akalu, T. Y.; Akanda, A. S.; Alahdab, F.; Al-Aly, Z.; Alam, S.; Alam, N.; Alamene, G. M.; Alanzi, T. M.; Albright, J.; Albujeer, A.; Alcalde-Rabanal, J. E.; Alebel, A.; Alemu, Z. A.; Ali, M.; Alijanzadeh, M.; Alipour, V.; Aljunid, S. M.; Almasi, A.; Almasi-Hashiani, A.; Al-Mekhlafi, H. M.; Altirkawi, K. A.; Alvis-Guzman, N.; Alvis-Zakzuk, N. J.; Amini, S.; Amit, A. M. L.; Amul, G. G. H.; Andrei, C. L.; Anjomshoa, M.; Ansariadi, A.; Antonio, C. A. T.; Antony, B.; Antriyandarti, E.; Arabloo, J.; Aref, H. M. A.; Aremu, O.; Armoon, B.; Arora, A.; Aryal, K. K.; Arzani, A.; Asadi-Aliabadi, M.; Asmelash, D.; Atalay, H. T.; Athari, S. M.; Athari, S. S.; Atre, S. R.; Ausloos, M.; Awasthi, S.; Awoke, N.; Quintanilla, B. P. A.; Ayano, G.; Ayanore, M. A.; Aynalem, Y. A.; Azari, S.; Azman, A. S.; Babaee, E.; Badawi, A.; Bagherzadeh, M.; Bakkannavar, S. M.; Balakrishnan, S.; Banach, M.; Banoub, J. A. M.; Barac, A.; Barboza, M. A.; Bärnighausen, T. W.; Basu, S.; Bay, V. D.; Bayati, M.; Bedi, N.; Beheshti, M.; Behzadifar, M.; Behzadifar, M.; Ramirez, D. F. B.; Bell, M. L.; Bennett, D. A.; Benzian, H.; Berbada, D. A.; Bernstein, R. S.; Bhat, A. G.; Bhattacharyya, K.; Bhaumik, S.; Bhutta, Z. A.; Bijani, A.; Bikbov, B.; Sayeed, M. S. B.; Biswas, R. K.; Bohlouli, S.; Boufous, S.; Brady, O. J.; Briko, A. N.; Briko, N. I.; Britton, G. B.; Brown, A.; Nagaraja, S. B.; Butt, Z. A.; Cámera, L. A.; Campos-Nonato, I. R.; Rincon, J. C. C.; Cano, J.; Car, J.; Cárdenas, R.; Carvalho, F.; Castañeda-Orjuela, C. A.; Castro, F.; Cerin, E.; Chalise, B.; Chattu, V. K.; Chin, K. L.; Christopher, D. J.; Chu, D.-T.; Cormier, N. M.; Costa, V. M.; Cromwell, E. A.; Dadi, A. F. F.; Dahiru, T.; Dahlawi, S. M. A.; Dandona, R.; Dandona, L.; Dang, A. K.; Daoud, F.; Darwesh, A. M.; Darwish, A. H.; Daryani, A.; Das, J. K.; Gupta, R. D.; Dash, A. P.; Dávila-Cervantes, C. A.; Weaver, N. D.; Hoz, F. P. D. la; Neve, J.-W. D.; Demissie, D. B.; Demoz, G. T.; Denova-Gutiérrez, E.; Deribe, K.; Desalew, A.; Dharmaratne, S. D.; Dhillon, P.; Dhimal, M.; Dhungana, G. P.; Diaz, D.; Dipeolu, I. O.; Do, H. T.; Dolecek, C.; Doyle, K. E.; Dubljanin, E.; Duraes, A. R.; Edinur, H. A.; Effiong, A.;

Eftekhari, A.; Nahas, N. E.; Zaki, M. E. S.; Tantawi, M. E.; Elhabashy, H. R.; El-Jaafary, S. I.; El-Khatib, Z.; Elkout, H.; Elsharkawy, A.; Enany, S.; Endalew, D. A.; Eshrati, B.; Eskandarieh, S.; Etemadi, A.; Ezekannagha, O.; Faraon, E. J. A.; Fareed, M.; Faro, A.; Farzadfar, F.; Fasil, A. F.; Fazlzadeh, M.; Feigin, V. L.; Fekadu, W.; Fentahun, N.; Fereshtehnejad, S.-M.; Fernandes, E.; Filip, I.; Fischer, F.; Flohr, C.; Foigt, N. A.; Folayan, M. O.; Foroutan, M.; Franklin, R. C.; Frostad, J. J.; Fukumoto, T.; Gad, M. M.; Garcia, G. M.; Gatotoh, A. M.; Gayesa, R. T.; Gebremedhin, K. B.; Geramo, Y. C. D.; Gesesew, H. A.; Gezae, K. E.; Ghashghaee, A.; Sherbaf, F. G.; Gill, T. K.; Gill, P. S.; Ginindza, T. G.; Girmay, A.; Gizaw, Z.; Goodridge, A.; Gopalani, S. V.; Goulart, B. N. G.; Goulart, A. C.; Grada, A.; Green, M. S.; Gubari, M. I. M.; Gugnani, H. C.; Guido, D.; Guimarães, R. A.; Guo, Y.; Gupta, R.; Gupta, R.; Ha, G. H.; Haagsma, J. A.; Hafezi-Nejad, N.; Haile, D. H.; Haile, M. T.; Hall, B. J.; Hamidi, S.; Handiso, D. W.; Haririan, H.; Hariyani, N.; Hasaballah, A. I.; Hasan, M. M.; Hasanzadeh, A.; Hassen, H. Y.; Hayelom, D. H.; Hegazy, M. I.; Heibati, B.; Heidari, B.; Hendrie, D.; Henok, A.; Herteliu, C.; Heydarpour, F.; Hidru, H. D. de; Hird, T. R.; Hoang, C. L.; Hollerich, G. I.; Hoogar, P.; Hossain, N.; Hosseinzadeh, M.; Househ, M.; Hu, G.; Humayun, A.; Hussain, S. A.; Hussen, M. A. A.; Ibitoye, S. E.; Ilesanmi, O. S.; Ilic, M. D.; Imani-Nasab, M. H.; Iqbal, U.; Irvani, S. S. N.; Islam, S. M. S.; Ivers, R. Q.; Iwu, C. J.; Jahanmehr, N.; Jakovljevic, M.; Jalali, A.; Jayatilleke, A. U.; Jenabi, E.; Jha, R. P.; Jha, V.; Ji, J. S.; Jonas, J. B.; Jozwiak, J. J.; Kabir, A.; Kabir, Z.; Kanchan, T.; Karch, A.; Karki, S.; Kasaeian, A.; Kasahun, G. G.; Kasaye, H. K.; Kassa, G. G.; Kassa, G. M.; Kayode, G. A.; Kebede, M. M.; Keiyoro, P. N.; Ketema, D. B.; Khader, Y. S.; Khafaie, M. A.; Khalid, N.; Khalilov, R.; Khan, E. A.; Khan, J.; Khan, M. N.; Khatab, K.; Khater, M. M.; Khater, A. M.; Khayamzadeh, M.; Khazaei, M.; Khosravi, M. H.; Khubchandani, J.; Kiadaliri, A.; Kim, Y. J.; Kimokoti, R. W.; Kisa, S.; Kisa, A.; Kochhar, S.; Kolola, T.; Komaki, H.; Kosen, S.; Koul, P. A.; Koyanagi, A.; Krishan, K.; Defo, B. K.; Kugbey, N.; Kumar, P.; Kumar, G. A.; Kumar, M.; Kusuma, D.; Vecchia, C. L.; Lacey, B.; Lal, A.; Lal, D. K.; Lam, H.; Lami, F. H.; Lansingh, V. C.; Lasrado, S.; Lebedev, G.; Lee, P. H.; LeGrand, K. E.; Leili, M.; Lenjebo, T. L.; Leshargie, C. T.; Levine, A. J.; Lewycka, S.; Li, S.; Linn, S.; Liu, S.; Lopez, J. C. F.; Lopukhov, P. D.; Razek, M. M. A. E.; Prasad, D. R. M.; Mahasha, P. W.; Mahotra, N. B.; Majeed, A.; Malekzadeh, R.; Malta, D. C.; Mamun, A. A.; Manafi, N.; Mansournia, M. A.; Mapoma, C. C.; Martinez, G.; Martini, S.; Martins-Melo, F. R.; Mathur, M. R.; Mayala, B. K.; Mazidi, M.; McAlinden, C.; Meharie, B. G.; Mehndiratta, M. M.; Nasab, E. M.; Mehta, K. M.; Mekonnen, T.; Mekonnen, T. C.; Meles, G. G.; Meles, H. G.; Memiah, P. T. N.; Memish, Z. A.; Mendoza, W.; Menezes, R. G.; Mereta, S. T.; Meretoja, T. J.; Mestrovic, T.; Metekiya, W. M.; Metekiya, W. M.; Miazgowski, B.; Miller, T. R.; Mini, G. K.; Mirrakhimov, E. M.; Moazen, B.; Mohajer, B.; Mohammad, Y.; Mohammad, D. K.; Mezerji, N. M. G.; Mohammadibakhsh, R.; Mohammed, S.; Mohammed, J. A.; Mohammed, H.; Mohebi, F.; Mokdad, A. H.; Moodley, Y.; Moradi, M.; Moradi, G.; Moradi-Joo, M.; Moraga, P.; Morales, L.; Mosapour, A.; Mosser, J. F.; Mouodi, S.; Mousavi, S. M.; Mozaffor, M.; Munro, S. B.; Muriithi, M. K.; Murray, C. J. L.; Musa, K. I.; Mustafa, G.; Muthupandian, S.; Naderi, M.; Nagarajan, A. J.; Naghavi, M.; Naik, G.; Nangia, V.; Nascimento, B. R.; Nazari, J.; Ndwandwe, D. E.; Negoi, I.; Netsere, H. B.; Ngunjiri, J. W.; Nguyen, C. T.; Nguyen, H. L. T.; Nguyen, Q. P.; Nigatu, S. G.; Ningrum, D. N. A.; Nnaji, C. A.; Nojomi, M.; Norheim, O. F.; Noubiap, J. J.; Oancea, B.; Ogbo, F. A.; Oh, I.-H.; Olagunju, A. T.; Olusanya, J. O.; Olusanya, B. O.; Onwujekwe, O. E.; Ortega-Altamirano, D. V.; Osarenotor, O.; Osei, F. B.; Owolabi, M. O.; A, M. P.; Padubidri, J. R.; Pakhale, S.; Pana, A.; Park, E.-K.; Patel, S. K.; Pathak, A.; Patle, A.; Paulos, K.; Pepito, V. C. F.; Perico, N.; Pervaiz, A.; Pescarini, J. M.; Pesudovs, K.; Pham, H. Q.; Pigott, D. M.; Pilgrim, T.; Pirsaheb, M.; Poljak, M.; Pollock, I.; Postma, M. J.; Pourmalek, F.; Pourshams, A.; Prada, S. I.; Preotescu, L.; Quintana, H.; Rabiee, N.; Rabiee, M.; Radfar, A.; Rafiei, A.; Rahim, F.; Rahimi, S.; Rahimi-Movaghar, V.; Rahman, M. A.; Rahman, M. H. U.; Rajati, F.; Ranabhat, C. L.; Rao, P. C.; Rasella, D.; Rath, G. K.; Rawaf, S.; Rawal, L.; Rawasia, W. F.;

Remuzzi, G.; Renjith, V.; Renzaho, A. M. N.; Resnikoff, S.; Riahi, S. M.; Ribeiro, A. I.; Rickard, J.; Roever, L.; Ronfani, L.; Rubagotti, E.; Rubino, S.; Saad, A. M.; Sabour, S.; Sadeghi, E.; Moghaddam, S. S.; Safari, Y.; Sagar, R.; Sahraian, M. A.; Sajadi, S. M.; Salahshoor, M. R.; Salam, N.; Saleem, A.; Salem, H.; Salem, M. R.; Salimi, Y.; Salimzadeh, H.; Samy, A. M.; Sanabria, J.; Santos, I. S.; Santric-Milicevic, M. M.; Jose, B. P. S.; Saraswathy, S. Y. I.; Sarrafzadegan, N.; Sartorius, B.; Sathian, B.; Sathish, T.; Satpathy, M.; Sawhney, M.; Sayyah, M.; Sbarra, A. N.; Schaeffer, L. E.; Schwebel, D. C.; Senbeta, A. M.; Senthilkumaran, S.; Sepanlou, S. G.; Serván-Mori, E.; Shafieesabet, A.; Shaheen, A. A.; Shahid, I.; Shaikh, M. A.; Shalash, A. S.; Shams-Beyranvand, M.; Shamsi, M.; Shamsizadeh, M.; Shannawaz, M.; Sharafi, K.; Sharma, R.; Sheikh, A.; Shetty, B. S. K.; Shiferaw, W. S.; Shigematsu, M.; Shin, J. I.; Shiri, R.; Shirkoohi, R.; Shivakumar, K. M.; Si, S.; Siabani, S.; Siddiqi, T. J.; Silva, D. A. S.; Singh, V.; Singh, N. P.; Singh, B. B. S.; Singh, J. A.; Singh, A.; Sinha, D. N.; Sisay, M. M.; Skiadaresi, E.; Smith, D. L.; Filho, A. M. S.; Sobhiyeh, M. R.; Sokhan, A.; Soriano, J. B.; Sorrie, M. B.; Soyiri, I. N.; Spurlock, E. E.; Sreeramareddy, C. T.; Sudaryanto, A.; Sufiyan, M. B.; Suleria, H. A. R.; Sykes, B. L.; Tabarés-Seisdedos, R.; Tabuchi, T.; Tadesse, D. B.; Tarigan, I. U.; Taye, B.; Tefera, Y. M.; Tehrani-Banihashemi, A.; Tekelemedhin, S. W.; Tekle, M. G.; Temsah, M.-H.; Tesfay, B. E.; Tesfay, F. H.; Tessema, Z. T.; Thankappan, K. R.; ThekkePurakkal, A. S.; Thomas, N.; Thompson, R. L.; Thomson, A. J.; Topor-Madry, R.; Tovani-Palone, M. R.; Traini, E.; Tran, B. X.; Tran, K. B.; Ullah, I.; Unnikrishnan, B.; Usman, M. S.; Uthman, O. A.; Uzochukwu, B. S. C.; Valdez, P. R.; Varughese, S.; Veisani, Y.; Violante, F. S.; Vollmer, S.; W/hawariat, F. G.; Waheed, Y.; Wallin, M. T.; Wang, Y.-P.; Wang, Y.; Wangdi, K.; Weiss, D. J.; Weldesamuel, G. T.; Werkneh, A. A.; Westerman, R.; Wiangkham, T.; Wiens, K. E.; Wijeratne, T.; Wiysonge, C. S.; Wolde, H. F.; Wondafrash, D. Z.; Wonde, T. E.; Worku, G. T.; Yadollahpour, A.; Jabbari, S. H. Y.; Yamada, T.; Yaseri, M.; Yatsuya, H.; Yeshaneh, A.; Yilma, M. T.; Yip, P.; Yisma, E.; Yonemoto, N.; Younis, M. Z.; Yousof, H.-A. S. A.; Yu, C.; Yusefzadeh, H.; Zadey, S.; Moghadam, T. Z.; Zaidi, Z.; Zaman, S. B.; Zamani, M.; Zandian, H.; Zar, H. J.; Zerfu, T. A.; Zhang, Y.; Ziapour, A.; Zodpey, S.; Zuniga, Y. M. H.; Hay, S. I.; Reiner, R. C. Mapping Geographical Inequalities in Access to Drinking Water and Sanitation Facilities in Low-Income and Middle-Income Countries, 2000–17. *The Lancet Global Health* **2020**, *8* (9), e1162–e1185. https://doi.org/10.1016/S2214-109X(20)30278-3.
